# Convergent TP53 loss and evolvability in cancer

**DOI:** 10.1186/s12862-023-02146-6

**Published:** 2023-09-25

**Authors:** Marcela Braga Mansur, Mel Greaves

**Affiliations:** Centre for Evolution and Cancer, The Institute of Cancer Research, ICR, London, UK

**Keywords:** convergence, *TP53*, hypoxia, drug resistance, stem cells, cancer

## Abstract

Cancer cell populations evolve by a stepwise process involving natural selection of the fittest variants within a tissue ecosystem context and as modified by therapy. Genomic scrutiny of patient samples reveals an extraordinary diversity of mutational profiles both between patients with similar cancers and within the cancer cell population of individual patients. Does this signify highly divergent evolutionary trajectories or are there repetitive and predictable patterns?

Major evolutionary innovations or adaptations in different species are frequently repeated, or convergent, reflecting both common selective pressures and constraints on optimal solutions. We argue this is true of evolving cancer cells, especially with respect to the *TP53* gene. Functional loss variants in *TP53* are the most common genetic change in cancer. We discuss the likely microenvironmental selective pressures involved and the profound impact this has on cell fitness, evolvability and probability of subsequent drug resistance.

One of the striking features of cancer that has emerged in the post 2000 genomic era is the extraordinary mutational diversity both between patients and within the cancer cell population of each individual patient [1–3]. It has illuminated why treatment response can be so variable and encouraged the idea of personalised medicine in which each patient’s cancer genomic profile impacts on choice of therapy.

This insight is both enlightening and very challenging. Cancers all develop by a classical Darwinian process of mutational diversification and natural selection within the ecosystem of the body’s tissues [4–6]. Are evolutionary trajectories of mutant clones of cells widely divergent or are there discernible patterns and predictability? Here we argue that loss of function mutations and deletions in the *TP53* gene are highly convergent and empower enhanced evolvability increasing the probability of metastasis and drug resistance.

## Convergence in evolution

The distinguished evolutionary biologist Stephen Jay Gould argued that if the tape of life could be re-run, then life in all its rich diversity would look very different and we probably would not be here [7]. His argument was based on the notion that adaptive changes in evolution are highly contingent, or dependent, on a sequence of variable and unpredictable circumstances. Others, including, in particular, Simon Conway Morris, champion the opposite view, namely that evolution by natural selection follows predictable and restricted paths and repeats itself from different starting points and in different species [8]. Evolution, it is forcefully argued, is highly convergent and this over-rides any rare contingencies. Run the tape again, counters Morris, and, déjà vu, the same thing happens and dominant *Homo sapiens* species takes centre stage.

Other investigators [9], take a balanced or nuanced view suggesting that the starting point and environmental circumstances are crucial. And, as a consequence, evolutionary trajectories and outcomes can be widely divergent and unique or repetitive, according to ecological circumstances.

There is no shortage of convincing examples of convergent evolution where an adaptive, phenotypic trait with clear fitness benefits has been ‘invented’ independently multiple times in different species [8, 9]. The ‘classic’ example is with eyes with light focusing lenses and photosensitive neuronal connections to a brain that have evolved multiple times [8]. Eyes in different species can look highly variable in architecture and number, but the functional anatomy of human and octopus’ eyes are remarkably similar [8]. And, interestingly, eyes that have evolved independently in different species use the same or very similar or derivative visual pigments (opsins) as in photosensitive prokaryotes [10]. Eye development across invertebrate and vertebrate species is orchestrated by the same DNA transcription regulating gene, *PAX6* [11]. This emphasises that independent or convergent evolution does not necessarily involve invention entirely from scratch. Rather, evolution is parsimonious and uses or repurposes what is already available.

The plethora of examples of evolutionary convergence has a simple rationale. This is that convergence is bound to happen because it reflects at a fundamental level how evolution by natural selection works. A repetitive evolutionary innovation reflects the optimal available adaptive solution to some common,or repeated, strong environmental challenge. And, crucially, the number of potential solutions is constrained by prior history of the players, their genetic and molecular circuits, anatomical features or what’s available as raw material. The laws of physics and chemistry also restraint options. Trees and mammals need to optimise respiratory, gaseous exchange. The best solution is to maximise surface area by a dense, branching architecture which is why trees and lungs look like inverted images of each other.

As further examples, consider the hydrodynamic challenge of swimming swiftly in water or the aerodynamic needs of flight. The laws of physics come into play, as well as biological capacities, limiting potentially available solutions. Which is why sharks, ichthyosaur reptiles and dolphins have much the same shapes and the wings of insects, birds and bats look similar [9]. Unsurprisingly, the fine details vary, reflecting the different starting points but, in principle, the adaptive solution is the same.

Convergent adaptation has been successfully field-tested [9], but more stringent experimental validation is possible under controlled laboratory conditions. The simplest, but most robust and persuasive, experiments are with bacterial microbes, in a culture flask, dividing rapidly, every 20 minutes. They can be subjected to a contrived environmental challenge, such as nutrient deprivation, and the outcome assessed over thousands of generations in a huge number of parallel experiments. Arguably, the most impressive of these experiments, was conducted by Rich Lenski over two decades or more [12]. It provided compelling evidence for convergence or repetitiveness of adaptive solutions reflected in cell shape, speed of division and metabolic rewiring.

Somatic cell evolution and diversification in cancer has broad parallels with adaptive variation and speciation of asexual unicellular protists [13, 14]. Assuming similar evolutionary principles apply then it might be anticipated that, depending on strength of selection and fitness advantage, or coefficient [15], that some cancer cell adaptive characteristics would be repetitively or convergently selected despite extensive genetic and phenotypic diversification. Identifying such underlying trends could have implications for the predictability of cancer progression, the probability of drug resistance and prognoses.

## Convergence in cancer: the natural experiment

Every year around 20 million people worldwide develop cancer [16]. That’s 20 million individual, but repeated, natural experiments in somatic cell evolution a year. The starting point – single-cell mutation within a human tissue ecosystem venue, is similar in every replicate. And then oncologists intervene to provide potent ‘artificial’ selective pressures with drugs, irradiation or immunotherapy [17]. This is a mega test for evolutionary convergence. In this context, the fact that metastatic disease is a very common destination in the natural history of cancer and drug resistance a highly prevalent response to therapy in advanced disease is itself a strong indication of evolutionary convergence [14].

The trajectory of travel for a clone of cancer cells, starting with a single mutant ancestor cell, is to expand population size, disperse from the site of origin and hijack the space and resources of other tissue ecosystems in the host. Unsurprisingly, this renegade journey is challenging and hazardous and there is evidence to suggest that most incipient malignant clones do not make it through [18]. At each stage of the way there are barriers or bottlenecks to overcome, including negative feedback signals restraining proliferation, formidable architectural roadblocks and a predatory immune system. And that’s only before oncologists bring their powerful weaponry into play. To succeed, cancer cells need to evolve by serial adaptation. They satisfy that requirement by genetic diversification and epigenic plasticity which provide novel, stable and adaptive phenotypes or traits.

Evolutionary potential is underpinned by mutational diversity, but natural selection is a test and filter for phenotypes and their fitness attributes, all assessed in the context of prevailing ecological pressures. Functionally relevant or adaptive phenotypic changes in cancer turn out to be repetitive and limited in number. Hanahan and Weinberg referred to these as ‘hallmark’ features of cancer [19]. These include metabolic switch to glycolysis and loss of signal pathway functions including avoidance of cell death or apoptosis signals. The number of consistent, hallmark features was initially suggested to be six but then increased to fourteen [20]. Some of these features are linked to genetic changes in cancer cells, others are epigenetic, reflecting plasticity of cellular phenotypes. Selection of signal pathway alteration rather than a property encoded by a single gene provides more options for convergent adaptation. But these convergent, phenotypic features of cancer cells are not drawn on a blank canvas or invented from scratch. Indeed, essentially all these features are inherent to normal cells but under tight regulation with expression dependent on cell type, time and place [13]. They may, for example, be expressed transiently, during embryogenesis, regeneration or wound healing. Their repetitive and stable expression in cancer is then a consequence of adaptation by deregulation. As in evolution in general, adaptive innovation that might seem mathematically very improbable can emerge, repeatedly, by modifying existing cellular circuitry [21]. But what does the genomics of cancers suggest?

## Convergence of genotypes

**Fig. 1:**
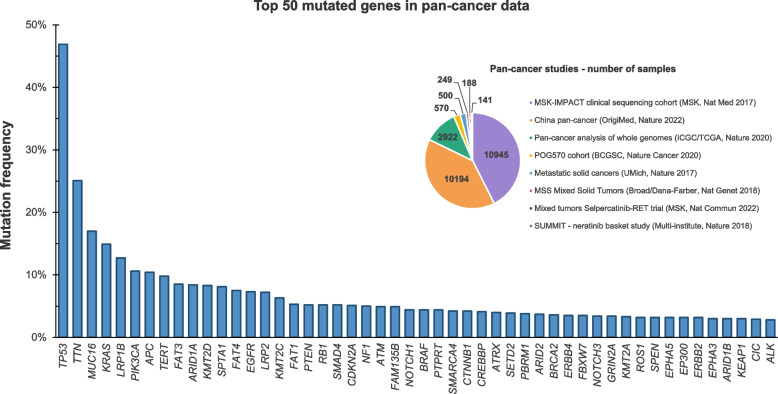
Recurrency of mutations in cancer showing *TP53* in pole position These data were generated by the cBioPortal for Cancer Genomics (https://www.cbioportal.org/) [23, 24]. We selected the PanCancer Studies database and included only the curated set of non-redundant studies in adults, which included 8 different studies with worldwide cases [25–32]. This dataset comprised 25,709 different pan-cancer samples specified by malignant cancer site (anatomical location or topography) and histology (morphology). The piechart shows the number of samples for each study included. Of note, the five most common cancer types were lung adenocarcinoma, colorectal adenocarcinoma, breast carcinoma, pancreatic adenocarcinoma and prostate adenocarcinoma and we displayed in the current figure the top 50 mutated genes. Sequencing data, from the 8 analysed studies, were obtained by using next-generation sequencing (NGS)/targeted sequencing, whole genome paired-end sequencing (WGS), and whole transcriptome/exome paired-end sequencing (WES). For further details about how the data was generated, curated and processed, please see original articles [25–32] and the cBioPortal webpage

The mutation count in individual cancers varies from one, or possibly, in rare cases none [22] to thousands. But the latter number mostly reflects genomic instability and the accumulation of many mutations that are synonymous base changes, neutral in function and considered as ‘passengers’. A smaller number (~ 100s) but still substantial subset of mutations and gross chromosomal changes are considered to be functionally relevant ‘drivers’ of malignancy. This view is endorsed by the recurrency of these mutations in different patients. But only a modest number of these mutations have high-level recurrency in, say, more than 5 to 10% of patients (Fig. 1). These include both activated oncogenes and tumour suppressor genes that either have mutational loss-of-function (LOF) or are deleted. High on the list of oncogene recurrency is *RAS* and other genes in the MAP kinase signalling pathway, and *RB1*, which is unsurprising, as these are key regulators of cell proliferation.

The application of machine learning and artificial intelligence (AI) to large datasets reveals reiterated patterns, sequences and combinations of mutations in different types of cancer [33]. This also reflects marked convergence and perhaps the restraints imposed by the way gene networks operate epistatically to impact on cell phenotype and behaviour. The distinctive cells-of-origin of different cancers also constrains and shapes mutational profiles [34].

In individual patients, interrogation of cancer cell genotypes at the single-cell level or in micro-dissected biopsies and representation of the data in the form of computed clonal, phylogenetic trees reveal that as subclones emerge, they independently accrue mutational changes in the same genes as well as distinct or divergent genetic changes [35–38]. This is indicative of intra-tumour convergent evolution though perhaps better regarded as parallel evolution, as it derives from a common founder cell [37].

These data indicate a significant degree of convergence exists at the genetic as well as phenotypic level. But frequently missing from these studies is consideration of the ecosystem context in which these mutants emerge, repetitively. The fitness benefits they provide are likely to be contingent upon the microenvironmental, adaptive landscape in which they are selected.

## Convergence of *TP53* mutation

The relevance of this contextual question is brought into sharp focus by a consideration of the single most common genetic alteration in cancer. Mutations and deletions in *TP53* are common genetic events in common adult cancers and hugely consequential for clonal progression of disease, signalling adverse clinical responses and poor outcomes [39, 40]. Figure 1 shows the composite results of 8 PanCancer mutational screenings involving more than 25,000 patients with mutations in *TP53* markedly more frequent than in an other gene.The pooled data is derived from multiple cancer types and at different stages, disguising variation. In some metastatic high-risk cancers (serous ovarian carcinoma, oesophageal adenocarcinoma and small cell lung carcinoma), *TP53* mutations are present at very high levels of 85–98% [25]. A comparison of primary versus metastatic samples reveals a substantial enrichment (up to ~ 50–100%) in the frequency of *TP53* mutations in secondary, metastatic lesions [26]. This observation concurs with the much earlier observation that progresion to high grade glioblastoma from prior low grade astrocytoma involves seletive outgrowth of rare *TP53* mutant cells present in the precursor malignancy [41]. Additionally, other genes in the TP53 signalling pathway may be mutated or with copy number alterations. Data in the COSMIC database [42] indicates that 15 of the estimated 67 TP53 pathway genes are recurrently altered including upstream *TP53* regulators, e.g. *MDM2* and *CDKN2A*, and downstream effectors, e.g. *CCDN1* and *PTEN* [40]. In some common virus associated cancers, TP53 function is blocked, as in cervical cancer, by HPV E6 oncoprotein which degrades TP53 protein [43]. In such cancers, TP53 functional status is critical but the gene itself is not then under selective pressure for mutational loss. Overall, the data supports the contention that the majority of advanced or metastatic cancer cell populations have defective TP53 function, indicative of highly convergent selection.

There are however some informative exceptions. A few rarer cancer types including the major subtype of childhood acute lymphoblastic leukaemia (ALL) and testicular seminoma are curable even though intrinsically malignant (-in the absence of treatment) and disseminated at diagnosis. These cancers are, in marked contrast to metastatic common adult cancers consistently *TP53* wild-type [44]. Elsewhere we argue that this distinction relates to differences in developmental origins and ecological pressures during clonal expansion [44].

**Fig. 2:**
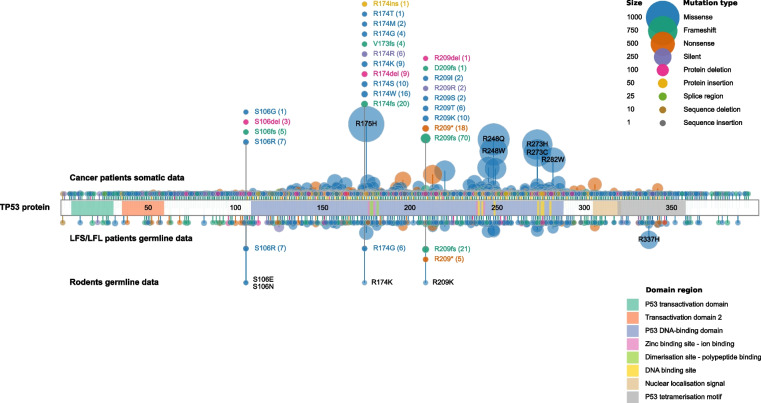
Map of *TP53* mutations in patients and Mole-rats We illustrate the mutational profile of *TP53* using an in-house developed data visualisation tool built with Python to display somatic and germline mutations from cancer patients (https://tp53.isb-cgc.org/) [45] and germline data from rodent species [47, 48]. For the rodent data, two previous publications showed four different germline mutations or variants found in three different rodent species: S104N-*Myospalax baileyi* (*M. b.*), S104E-*Microtus oeconomus* (*M. o.*), R172K and R207K-*Spalax judaei* (*S. j.*) [47, 48]. In human, these *TP53* variants correspond to S106N, S106E, R174K and R209K mutations, respectively. GRCh37/hg19 was used as our genome reference and NM_000546/NP_000537 as reference sequences for mutation annotations and protein domains. For the patient data, we included 4,299 samples from cases diagnosed with Li-Fraumeni Syndrome (LFS) and Li-Fraumeni-like Syndrome (LFL) (germline mutations) and 29,656 samples from general cancer patients (somatic mutations). We have filtered the database to only include samples with confirmed germline or somatic mutational status and with available genomic mutation annotation (GRCh37/hg19). The mutations are represented by discs at the codon position. The disc sizes and their distance from TP53 protein scheme are both proportional to the number of mutations. The most frequent alterations are annotated within each disc. The discs located above the protein scheme represent the somatic data for cancer patients, the ones immediately below refer to the germline mutations found in the LFS and LFL cases and the rodent data is displayed at the bottom of the plot. Mutations are coloured according to their effect (missense, frameshift-red, nonsense, silent, etc.) and TP53 protein structure is coloured highlighting its main domains (NP_000537). Even though, they are not frequent enough to be automatically highlighted (size of the discs and distance from TP53 protein scheme), we decided to display the ‘shared’ mutations between rodent and human cancer data for clarity purposes

Somatic *TP53* mutations in cancer are scattered throughout the gene but the critical functional domains for DNA binding and transactivation are hotspots (Fig. 2, The *TP53* Database R20, July 2019: https://tp53.isb-cgc.org) [45]. Some specific single nucleotide variants (SNVs) have been detected in hundreds if not thousands of individual patients (Fig. 2). Li-Fraumeni syndrome (LFS) patients with high cancer risk have germline *TP53* mutations similar to those acquired somatically (Fig. 2). Conversely to LFS, elephant species have evolved multiple copies of *TP53* which may contribute to their low cancer rates especially in relation to their size (and cell numbers at risk) and longevity [46].

Most *TP53* mutations are coupled with deletion of the wild-type allele resulting in loss of TP53’s DNA binding and normal pan-regulatory activities (see further below). A minority of *TP53* mutations are activating [49], but some at least are dominant negative also resulting in LOF.

Evolutionary adaptations are usually viewed as gains of novel functions but bacterial studies have revealed that LOF can, under some circumstances, rewire regulatory networks enabling a fitness advantage [50].

The very high degree of recurrency of *TP53* mutation in cancer is difficult to explain except by convergent natural selection. But this begs the question of defining adaptive or fitness advantages and the consistent selective pressures involved. Ascribing evolutionary traits adaptations to particular ecological variables is recognised as difficult, with speculations, in Gould’s words, as ‘just so’ stories. Any apparent adaptations could, in theory, be serendipitous or co-selected (or piggy backed) with some other feature. In the case of cancer and TP53, we suggest the following four criteria or tests for convergent selection:

- Highly recurrent presence in cancer, i.e. indicative of repeated independent selection (as references above and in Figs. 1 and 2);
- Definable fitness benefits for cells with TP53 loss, i.e. indicative of adaptive significance;
- Strong association with one or more prevalent microenvironmental features of cancer, i.e. identification of possible selective pressures;
- Demonstration that a candidate selective pressure when applied experimentally in a model system results in selection of *TP53* mutant cells, i.e. experimental, functional validation of adaptive logic.

Here we argue that TP53 loss in cancer satisfies all four criteria. The same applies to cancer drug resistance (see below).

### Definable fitness benefits

The *TP53* gene was an early evolutionary innovation in unicellular protists functioning as a cell stress detector [51] and plays a critical ‘Guardian of the genome’ role in multicellular animal development and in germline maintenance, eliminating damaged cells by apoptosis [52]. A possibly incidental but crucial benefit of these surveillance and restraint functions of TP53 is to reduce risk of cancerous cell transformation and disease progression. Hence the label tumour suppressor [51, 52].

Cancer clone evolution involves transit though bottlenecks that may activate TP53 which then restrains cell proliferation, enabling DNA repair or eliciting cell death. These bottlenecks include the proliferative stress imposed by potent oncogenes [52] and DNA damaging genotoxic exposures [53]. But arguably the most consistent bottleneck is intra-tumoral hypoxia and acidosis [52, 54, 55] which results in large scale cell death by a TP53-dependent mechanism. The fitness benefits to cancer cells of TP53 functional loss in these settings include not only cell survival, but tolerance of oncogenic drive [52, 56] and genetic instability. Model studies reveal that sequential genomic alterations including deletions, ploidy changes and copy number gains arise predictably, in a deterministic fashion, after TP53 LOF [57, 58].

**Fig. 3:**
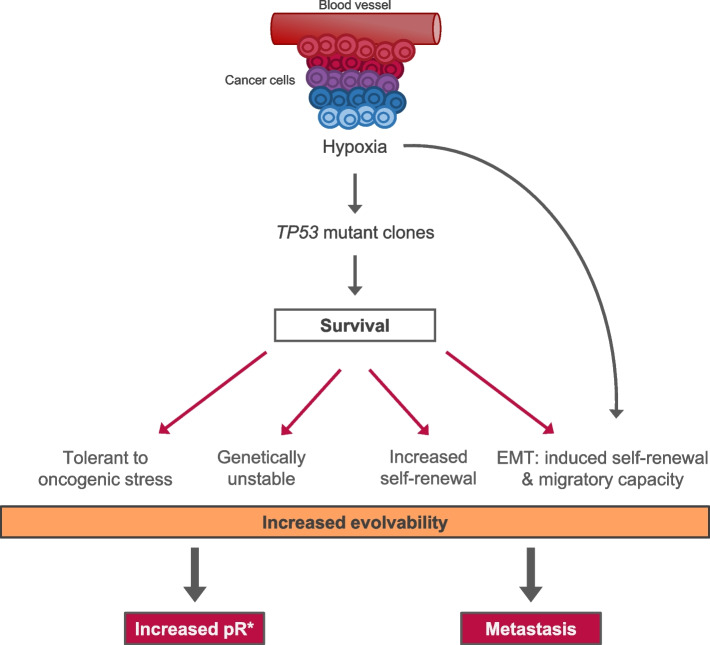
Surviving hypoxia: *TP53* mutant selection and evolvability Figure illustrates multiple fitness impacts in cells surviving hypoxic intra-tumour environments via TP53 LOF. These phenotypic features compound to increase adaptive evolvability enabling both metastasis and drug resistance. EMT, epithelial-mesenchymal transition. The curved line is to indicate that EMT is a response to hypoxia (see text for explanation), but is only likely to happen if cells can survive hypoxia/acidosis-associated cell death. Which in turn is much more probable if TP53 signalling is aborted. Note that ocogenic or genotoxic stress can also select for TP53 LOF in the absence of hypoxia and with the same fitness benefits. However, the overall impact on evolvability will be less in the absence of hypoxia-driven EMT

Other very consequential changes in *TP53* mutants involve self-renewal or stem cell-like functions. *TP53* normally imposes quiescence, restraining self-renewal of cells [59–61]. Loss of this restraint via *TP53* mutation and deletion therefore releases the self-renewal activity of cells [59, 62, 63]. Cells with potent self-renewal can be considered as the critical units of selection in cancer, responsible for progression of disease, metastatic colonisation and drug resistant recurrence [13, 64]. This important gear change in cancer clone expansion is further accelerated by an adaptive, epigenetic response in TP53-null cancer cells surviving hypoxia. The hypoxic environment in tumours resembles developmental mesenchymal niches with low oxygen levels. In response, epithelial cancer cells in hypoxic zones undergo epithelial-mesenchymal transition (EMT) with activated stem cell transcriptional and migratory programmes, enabling both clonal expansion and metastasis [65, 66]. Collectively, the impact of TP53 loss on cellular fitness is huge and provides for enhanced evolvability [67]: more self-renewing cells in play with more genetic instability and mutational diversity, providing a richer substate for adaptations and selection (Fig. 3).

### Association between hypoxia and *TP53* mutant selection

Although the pressures of oncogenic, proliferative stress and genotoxic exposures contribute significantly to the selection of TP53 LOF and signal pathway variants, we suggest that the most prevalent and potent pressure derives from metabolic changes and associated hypoxia and acidosis within the tumour microenvironment. The extent of intra-tumour hypoxia depends upon vascularisation, tumour volumes and the diffusion properties of oxygen [68]. It is, therefore, variable between different patients with similar diagnoses and variable within individual tumours. But, overall, there is a strong correlation in multiple different cancers between the extent of hypoxia and frequency of *TP53* mutations [69, 70]. These associations are consistent with the argument that hypoxia selects for *TP53* mutants but do not provide evidence of causality.

The likely adaptive logic of *TP53* mutant selection in the context of highly prevalent ecosystem hypoxia is reinforced by remarkable example involving germline *TP53* variants in animal species. Species of Mole-rats within the family *Spalacidae* live underground or at high altitudes where they face severe environmental challenges with very oxygen levels (~ 10% of sea level above ground levels) associated high CO_2_ and acidity and, at high altitude, cold. The survival and success of these rodents, coupled with their remarkable longevity (> 20 years), has required multiple adaptations [47] including changes to the germline sequence of *TP53* and to the function of the encoded protein. Three species investigated in Israel and China have independently, or convergently, evolved germline mutations in *TP53* that mirror those found in cancer cells (Fig. 2) [47, 48]. All four mutations identified in the rodents have comparable counterparts in somatic variants in cancer cells (exact same amino acid position for all four, but only identical for R174K and R209K) and in LFS cases (exact same amino acid position for all four, but different amino acid changes) harbouring germline *TP53* mutations (see Fig. 2). Functionally, the Mole-rat *TP53* variants fail to activate apoptosis but, in common with some cancer cells somatic *TP53* mutations, they can still induce cell cycle arrest.

The mutation S104E (corresponding to S106E in humans), found in *Mycrotus oeconomus* (*M. o.*), is also present in four species of fish and the squid *Loligo forbesii* (*L. f.*) that likewise live in hypoxic environments [48]. Although lacking validating experimental evidence, the authors of these reports plausibly speculate that these sequence changes to germline *TP53* reflect adaptations to a sustained hypoxic environment or associated high CO_2_ and acidosis [47, 48]. If correct, then It is extraordinary that the adaptive, TP53-based adaptive solution to environmental hypoxia should be convergent between animal species and cancer cells.

Given the loss of TP53-dependent apoptosis status of the cells in these Mole-rat species, coupled with their longevity, they might be expected to be very cancer prone. But the opposite appears to be the case as they appear to suffer little or no cancer and, furthermore, their cells are resistant to experimentally induced cancer that is effective in other rodent species [71]. In contrast, germline mutations in *TP53* in humans with the LFS (Fig. 2) carry a high (~ 80%) risk of cancer [72]. These data imply the Mole-rat species have evolved compensatory adaptations to counteract a high intrinsic risk of cancer from mutant *TP53* and several potential mechanisms have been identified [71, 73]. The residual capacity of mutant *TP53* to induce cell cycle arrest and senescence in response to oncogenic and genotoxic stress is also likely to be relevant to low cancer risk [74].

### Functional validation

More direct evidence that hypoxia provides potent selective pressure for TP53 loss comes from experimental models. The imposition of metabolic stress and hypoxia on tumour cells and some normal cell populations demonstrably favours survival of TP53 loss mutants [75–77]. *TP53* mutant cancer cells selectively survive in the hypoxic and apoptotic cores of tumour organoids [78].

## *TP53* selection and enhanced evolvability of resistance

**Fig. 4:**
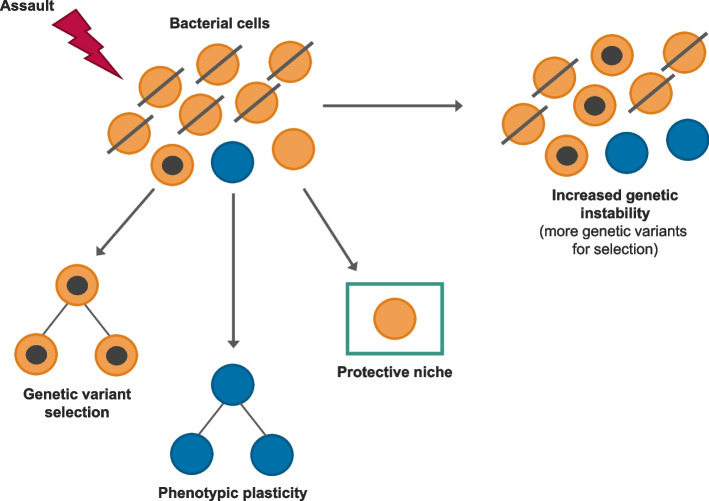
Evolutionarily ancient mechanisms of resistance in bacteria The three generic and evolutionarily conserved mechanims of escape are illustrated with black dots in cells representing mutations underpinning resistance. Phenotypic plasticity includes dormancy or proliferative quiescence as well as rapid adaptability of intracellular signalling networks

Once cancers disseminate or, at grade 4, they become very difficult to eradicate. Survival times have improved, but most of these cancers are incurable [44]. And the barrier is not the lack of innovative and well-targeted therapies, but the consistent emergence of drug resistance. This can be seen as a very convergent, adaptive response to the same or similar and potent selective pressure albeit iatrogenic or artificial [14, 17, 79]. Adaptive traits enabling cellular survival in adverse environments will have been a necessary early evolutionary, prokaryote innovation, and we see this mirrored in the adaptive tactics used by bacterial cells (Fig. 4) [80]. These cellular traits are highly conserved and the same generic mechanisms to resistance are available for selection in cancer cells. So, unsurprisingly, they are called into play in a highly repetitive or convergent fashion in the face of potentially lethal therapeutic challenge [81, 82].

In the case of highly targeted drugs, the nature of the resistance mechanism repetitively selected in different patients mirrors the target specificity of the drug applied [79, 83, 84], similarly in resistance to immunotherapy via immunoediting [85], demonstrating the adaptive logic of resistance selection. As in earlier bacterial models, multiple parallel experiments with drug exposure of barcoded cancer cells in vitro [86] reveals the deterministic, repetitive nature of resistance. These data satisfy the functional criterion for convergent evolution for drug resistance.

## Conclusions

*TP53* mutations and/or deletions and emergent drug resistance are, arguably, the most convergent or consistent features of malignant clones and hugely consequential for patients and clinical outcome. It is, therefore, of some interest that one adaptations facilitates the other. Their repetitiveness, or evolutionary convergence, reflects both the strong and consistent selective pressures and the very substantial cellular fitness benefits.

The adverse association of *TP53* mutation and therapeutic response and clinical outcome is striking and consistently observed in multiple cancer types [39, 40, 87, 88]. This reflects the major impact of *TP53* loss on cancer cell resilience and adaptability in the face of therapeutic challenge. Absence of TP53 function decreases intrinsic sensitivity to genotoxic drugs, irradiation and possibly immunotherapy also, which kill cells primarily via TP53-dependent apoptosis. And, at the same time, cancer cells with TP53 benefit from enhanced evolvability, which increases both the numbers of drug selectable self-renewing cancer cells and their mutational genetic diversity, tipping the numbers game, and probability, heavily in favour of resistance (Fig. 3).

The major clinical challenge in cancer—drug resistance—is a consequence of the consistent ecological pressures that elicit convergent evolution. TP53 pathway LOF is not the only or exclusive clonal trajectory in all of the millions of patients who develop metastatic disease and drug resistance. Other very recurrent mutations may drive disease progression to the same endpoint. This is likely to include other mutations enriched in the context of hypoxia, including *MYC* and *PTEN* [69, 70], the latter being in the TP53 pathway, as well as Ras-MAP kinase signalling. But the predominant and accelerated evolutionary trajectory, enabled by *TP53* loss, is exceptional and the cancer cells’ equivalent of acquiring wings.

How then to clip those wings? Elsewhere [44], we argue that the predictability of an adverse evolutionary trajectory provides a strong endorsement for a focus on prevention and early diagnosis and intervention, i.e. before the wings unfold. Therapeutic intervention in this ‘early’ context might include targeting the hypoxic microenvironment [89] or stem cells [90, 91].

There is some optimism that ‘*TP53* wings’ might be clipped by targeting mutant *TP53* or rescuing wild-type function [92–94] but this remains challenging to deliver in vivo. An alternative strategy for treating advanced disease is to restrain or slow down rather than attempt elimination, by adaptive therapy [95] or clonal steering by sequential drug exposure [86], both using evolutionary parameters as a real-time guide.

The evolutionary resilience of advanced cancer, consistently and convergently empowered by TP53 loss, is arguably the biggest barrier to therapeutic cure or control.

## Data Availability

The *TP53* mutational data used in our Fig. 2 is publicly available on The *TP53 *[45] database  website https://tp53.isb-cgc.org/ (patients) and in the references [47, 48] (rodents). The sequencing data used in our Fig. 1 is available on cBioPortal for Cancer Genomics (https://www.cbioportal.org/)  [23, 24] and detailed information related to these data is available in the original articles [25, 26, 27, 28, 29, 30, 31, 32].
